# Caffeic acid phenethyl ester protects 661W cells from H_2_O_2_-mediated cell death and enhances electroretinography response in dim-reared albino rats

**Published:** 2012-05-30

**Authors:** Hui Chen, Julie-Thu A. Tran, Robert E. Anderson, Md Nawajes A. Mandal

**Affiliations:** 1 Department of Ophthalmology, University of Oklahoma Health Sciences Center, Oklahoma City, OK; 2 Dean A. McGee Eye Institute, Oklahoma City, OK; 3 Department of Ophthalmology, Sichuan Academy of Medical Sciences & Sichuan Provincial People’s Hospital, Chengdu, Sichuan, China; 4 Department of Cell Biology, University of Oklahoma Health Sciences Center, Oklahoma City, OK

## Abstract

**Purpose:**

Caffeic acid phenethyl ester (CAPE), an active component of honeybee propolis, has a wide range of beneficial properties. The purpose of this study was to test the protective role of CAPE in 661W cells (in vitro) against H_2_O_2_-mediated cell death and in albino rats (in vivo) against various light conditions.

**Methods:**

The 661W cells were pretreated with CAPE and then stressed with H_2_O_2_. Cell death was measured with lactate dehydrogenase (LDH) release assay, and mRNA and proteins were analyzed. Sprague Dawley rats were raised on either a control or CAPE (0.02%) diet and exposed to various light conditions for short or long periods. Retinal histology, mRNA, protein, lipid composition, and retinal function by electroretinography (ERG) were measured at the end of feeding.

**Results:**

Pretreatment of 661W cells with CAPE reduced H_2_O_2_-mediated cell death in a dose-dependent manner and induced expression of heme oxygenase-1 (*Ho1*). Albino rats fed with CAPE had greater expression of *Ho1* and intercellular adhesion molecule 1 (*Icam1*), less expression of FOS-like antigen (*Fosl*) and lipoxygenase 12 (*Lox12*) genes in the retina, less translocation of nuclear factor kappaB protein to the nucleus, and a lower molar ratio of n-3 polyunsaturated fatty acids. Further, the ERGs of the retinas of CAPE-fed rats were significantly higher than those of the control-fed rats when raised in dim light.

**Conclusions:**

CAPE can activate the antioxidative gene expression pathway in retinal cells in vitro and in vivo. Feeding CAPE to albino rats can enhance ERG responses and change the lipid profile in the rats’ retinas.

## Introduction

Retinal degenerations are a heterogeneous group of diseases in which the light-sensitive photoreceptor cells and their supporting retinal pigment epithelium (RPE) cells die irreversibly. Mutations in as many as 150 genes have been found for the monogenic forms of these diseases. A complex inheritance pattern and environmental factors, such as exposure to light, combine for the complex etiology as in the cases of age-related macular degeneration [1-3]. Accumulating evidence suggests that excessive exposure to white light [4-6], oxidative damage (free radicals) [7-10], and inflammatory stresses [7,11] are some of the common pathogenic factors in the development of age-related macular degeneration [7,12] and progression of retinitis pigmentosa [13].

Caffeic acid phenethyl ester (CAPE) is an active component of honeybee propolis. CAPE has been used in Asian and Ayurvedic folk medicine since ancient times [14]. In the past three decades, several studies have shown that CAPE has a wide range of beneficial properties, including antioxidant, anti-inflammatory, antiproliferative, antiviral, antibacterial, and immunomodulatory properties [15-18]. The prominent protective property of CAPE makes it a potential therapeutic compound against damage for the heart [19-21], kidney [22-24], and other tissues or organs [25,26]. Recently, CAPE has been found to have a protective role in neurons or neural tissues. CAPE can block apoptosis in cerebellar granule cells [27], suppress ischemia-reperfusion-induced cerebral injury [28] and spinal cord ischemia/reperfusion injury [29], and prevent various toxin-induced neurodegeneration or neurotoxicity [30-34].

Since CAPE has well known anti-inflammatory and antioxidant properties, we hypothesize that CAPE may be able to protect retinal cells against oxidative and inflammatory stress-induced damage and can be used as an augmentative therapy for preventing or delaying the onset of retinal degeneration in humans. In this study, we used the H_2_O_2_-mediated cell death model of mouse photoreceptor-derived 661W cells and acute or chronic bright light-induced retinal degeneration models of albino Sprague Dawley (SD) rats to test the protective role of CAPE in vitro and in vivo.

## Methods

### Cell culture

Mouse photoreceptor-derived 661W cells [35] were kindly provided by Dr. Muayyad Al-Ubaidi (University of Oklahoma Health Sciences Center, Oklahoma City, OK). The 661W cells were maintained in Dulbecco's modified Eagle's medium (DMEM; Invitrogen, Carlsbad, CA). The medium contained 10% fetal bovine serum, 1 mM sodium pyruvate, 100 units/ml penicillin, and 100 μg/ml streptomycin. The cells were grown in 5% CO_2_, and 95% humidity at 37 °C.

### Cell viability assays

The 661W cells (4×10^6^) were cultured for 24 h in 10 ml medium in 10-cm plates. To test the cytoprotective effect of CAPE against H_2_O_2_-mediated cell death, the cells were pretreated for 3 h with 1 to 20 μM of CAPE (Cat# Q-2305; Bachem Americas Inc., Torrance, CA). The cells were then washed. Fresh medium was added to the cells, and 3 h later the cells were stressed with 1 mM of H_2_O_2_ (Sigma, St. Louis, MO) for another 6 h. Cell viability was determined indirectly by measuring the released lactate dehydrogenase (LDH) in the medium by using a commercial kit from Promega (CytoTox-ONE Homogenous Membrane Integrity Assay kit; Madison, WI) following the manufacturer's instructions. For the LDH release assay, the fluorescence measure of the release of LDH from the cells with a damaged membrane was calculated by subtracting the culture medium background, and the cell viability (%) was calculated from 100% viability (mean value of untreated cells) to 0% viability (mean value of 2% Triton X-100-treated cells, Sigma, St. Louis, MO). To isolate the RNA and proteins, 661W cells were scrapped from the 10-cm dishes, washed twice with RNase-free ice-cold phosphate-buffered saline (PBS), and collected with centrifugation. The cell pellets were stored at −80 °C until the RNA and proteins were extracted.

### Animal care

All procedures were performed according to the Association for Research in Vision and Ophthalmology Statement for the Use of Animals in Ophthalmic and Vision Research and the University of Oklahoma Health Sciences Center (OUHSC) Guidelines for Animals in Research. All protocols were reviewed and approved by the Institutional Animal Care and Use Committees of the OUHSC and the Dean A. McGee Eye Institute.

### Dietary supplementation of caffeic acid phenethyl ester and the design for light exposure to retina

#### Acute light stress and short-term dim light exposure

This study was performed by feeding six-week-old rats on either CAPE or control diets (n=8 in each group) in cyclic dim light (DL: 50 lux, 7 AM to 7 PM), and then exposing them to acute light stress. Briefly, pelleted CAPE and control diets were prepared by Dyets Inc. (Bethlehem, PA). Rats were housed four to a cage, and the diets were supplied on top of the cage cover. Water was provided ad libitum. SD rats were fed either with the AIN-93G diet (Control diet: Cont) or the AIN-93G diet supplemented with 0.02% CAPE (w/w; Bachem Americas Inc.; CAPE diet: CAPE). Based on published literature, to supply 10–20 mg/kg/day CAPE, the CAPE concentration was 0.02% in the diet [36-41]. When administered intravenously, 1–10 mg/kg CAPE has been shown to cross the blood–brain barrier and protect the rat brain after transient focal cerebral ischemia in a dose-dependent manner [42]; we speculated dietary CAPE can also be absorbed, transported through blood, and can reach the retina by crossing the blood–retinal barrier. After two weeks of feeding, two groups (CAPE and Cont) were kept in cyclic dim light; the other two groups (CAPE and Cont) were exposed to intense bright light at 2,700 lux for 6 h (acute light stress group). After intense light exposure, these groups were returned to their native dim cyclic light and fed their respective diets. Retinal function was measured with electroretinography (ERG) after one week of light damage. Then the rats were euthanized with CO_2_ from a compressed gas tank in an euthanization chamber followed by cervical dislocation, and their eyeballs were harvested for retinal histological analysis.

#### Chronic (long-term) dim or bright light exposure

Three-week-old (immediately after weaning) rats (12 rats per group) were fed either with an AIN-93G diet (Cont) or an AIN-93G diet supplemented with 0.02% CAPE (CAPE) in cyclic dim light (DL: 50 lux, 7 AM to 7 PM) for eight weeks. Additional three-week-old rats (12 rats per group) were fed with either the Cont diet or the CAPE diet and exposed to cyclic bright light (BL: 200 lux, 7 AM to 7 PM) for eight weeks to test the effect of CAPE on rat retinas by raising the rats in higher intensity cyclic light. Retinal function was measured at the end of eight weeks without any further light treatment. Immediately after the ERG was measured, the rats were euthanized, the right eyeballs were processed for quantitative morphology, and the neural retina from the left eye was harvested and stored in −80 °C for RNA, protein extraction, and fatty acid analysis.

### Electroretinography

Flash ERGs were recorded with an ERG recording system (Espion E2 ERG System; Diagnosys, Lowell, MA). Rats were maintained in total darkness overnight and prepared for ERG recording in dim red light. Each rat was anesthetized with ketamine (120 mg/kg bodyweight intramuscularly) and xylazine (6 mg/kg bodyweight, intramuscularly). One drop of 10% phenylephrine was applied to the cornea to dilate the pupil, and one drop of 0.5% proparacaine HCl was applied for local anesthesia. A reference electrode was positioned in the mouth and a ground electrode on the foot, and the rat was placed inside a Ganzfeld illuminating sphere with a gold electrode placed on the cornea. Four increasing strobe flashes were used starting at an intensity of −2.3 followed by −1.3, 0.7, and 2.7 log cd.s/m^2^, and the ERG responses from both eyes were recorded to assess rod photoreceptor function (scotopic ERG). To evaluate cone function (photopic ERG), a strobe flash stimulus (3.7 log cd.s/m^2^) was presented to dilated, light-adapted (5 min at 2.0 log cd.s/m^2^) rats. The amplitude of the A-wave was measured from the prestimulus baseline to the A-wave trough. The amplitude of the B-wave was measured from the trough of the A-wave to the peak of the B-wave.

### Measurement of the outer nuclear layer thickness

After the ERG was recorded, the animals were euthanized with CO_2_ asphyxiation in an euthanization chamber followed by cervical dislocation, and eyeballs were removed, fixed, and embedded in paraffin. Sections (5 μm thick) were taken through the optic nerve head along the vertical meridian to compare all regions of the retina in the superior and inferior hemispheres. In each hemisphere, the outer nuclear layer (ONL) thickness was measured at 480-μm intervals in seven defined areas, starting at the optic nerve head and extending toward the superior and inferior ora serrata. In addition, the mean ONL thickness was calculated for the inferior and the superior regions of each retina.

### Retinal fatty acid analysis

Whole neural retinas were harvested from rats fed either the Cont or CAPE diet and frozen immediately in liquid nitrogen. Retinal lipids were subsequently extracted with a modified Bligh–Dyer technique [43] in the presence of internal standards. In brief, the lipids from one retina were extracted in a Teflon/glass homogenizer using 2 ml of methanol/chloroform (1:1, v/v) with phase separation with the addition of 1.5 ml of saline. The methanol/chloroform mixture contained the following internal standards: di-14:0 phosphatidylethanolamine (PE; 23.6 nM), di-17:0 PE (23.6 nM), di-20:0 phosphatidylcholine (PC; 11.8 nM), and di-14:0 phosphatidylserine (PS; 1.8 nM). Lipids were extracted twice from the retinas, and the pooled chloroform layers were washed with the Folch theoretical upper phase before the solvent evaporated under a nitrogen stream and the layers were resuspended in chloroform [44].

For fatty acid composition analysis, fatty acid methyl esters were prepared from the total retinal lipid extracts by subjecting them to strong acid hydrolysis (16.6% HCL in methanol at 75 °C overnight) as described by Agbaga et al. [44]. The fatty acid methyl esters were separated from other sterols with thin layer chromatography and analyzed with a gas chromatography-flame ionization detector (Agilent Technologies, Santa Clara, CA) as described in Ford et al. [45].

### RNA isolation, cDNA synthesis, and quantitative reverse-transcriptase polymerase chain reaction

RNA was isolated and purified from frozen cultured 661W cells and rat retinas using the PureLink Micro-to-Midi Total RNA Purification System from Invitrogen following the manufacturer's protocol. Equal quantities (1.0 μg) of total RNA from each tissue were converted to first-strand cDNA using SuperScript III First-Strand Synthesis SuperMix (Invitrogen) for reverse-transcriptase polymerase chain reaction (RT–PCR). First-strand cDNA was used for quantitative reverse-transcriptase PCR (qRT–PCR). Primers for qRT–PCR were designed in such a way that they spanned at least one intron, which eliminated the chance of amplification from residual genomic DNA contamination. The primer sequences are provided (Table 1). Quantitative PCR and melt-curve analyses were performed using iQ SYBR Green Supermix (Bio-Rad, Hercules, CA) and an iCycler machine (Bio-Rad). The relative quantities of the expression of the genes of interest in different samples were calculated with the comparative *C*_t_ (threshold cycle) value method [46].

**Table 1:** Sequence of the primers used for RT–PCR.

**Gene**	**Forward Primer (5′-3′)**	**Reverse Primer (5′-3′)**
*Ho1*	TCTATCGTGCTCGCATGAAC	CTGTCTGTGAGGGACTCTGG
*Icam1*	TCAAACGGGAGATGAATGGT	AGTTTTAGGGCCTCCTCCTG
*Thx1*	GTGTGGACCTTGCAAAATGA	CCCAACCTTTTGACCCTTTT
*Nrf2*	AGGACATGGAGCAAGTTTGG	TCCTCAAAACCATGAAGGAA
*Cxcl1*	TAGCCACACTCAAGAATGGT	CTCCATTACTTGGGGACACC
*Lox5*	ACCTGACGGTGGTGATCTTC	GGCCACGGTCTGGTAGAGTA
*Lox12*	GCACAGCTCTGCCATTTCCT	GATGGTCATCTGCAGACAGG
*NfKb1*	ACAGATGGGCTACACAGAGG	TGTCTCCACACCACTGTCAC
*EGR1*	CTACGAGCACCTGACCACAG	AGGCCACTGACTAGGCTGAA
*iNos*	AGGTGCACACAGGCTACTCC	GGCCACCAGCTTCTTCAA
*Tnf-a*	CTCAAAACTCGAGTGACAAGC	GTGGGTGAGGAGCACGTAGT
*Fosl1*	AGAGCGGAACAAGCTAGCAG	CAAGTACGGGTCCTGGAGAA
*C-fos*	GAAACGGAGAATCCGAAGG	TGGGCTGCCAAAATAAACTC
*Catalase*	GCGCTTCAACAGTGCTAATG	AGGGTGGACGTCAGTGAAAT
*Cox2*	GGCACCAATGATACTGAAGC	TCAGAGCATTGGCCATAGAA
*IL6*	TGTGCAATGGCAATTCTGAT	GGAAGTTGGGGTAGGAAGGA
*Ccl2*	GTTAATGCCCCACTCACCTG	TTCCTTATTGGGGTCAGCAC
*Hgprt1*	CTTTGCTGACCTGCTGGATTAC	TTGGGGCTGTACTGCTTAACC
*RPL19*	GGGAAGAGGAAGGGTACTGC	GGACGCTTCATTTCTTGGTC

### Western blotting

Whole-cell lysates from the 661W cells and the retinal extracts were prepared for western blotting by sonicating in T-PER reagent (Pierce, Rockford, IL) containing a protease inhibitor cocktail (Roche, Indianapolis, IN) and then centrifuging at 10,000× g for 15 min at 4 °C to collect the supernatants. After the protein concentrations were determined using BCA reagent (Pierce), equal aliquots (20–30 μg) of protein samples were applied to 10% sodium dodecyl sulfate polyacrylamide gels (Invitrogen) and electrophoretically separated. Resolved proteins were electrophoretically transferred to nitrocellulose membranes (Bio-Rad) and blocked with 5% nonfat dry milk for 1 h at room temperature. The membranes were incubated with anti-HO-1 (1:1,000; R&D Systems, Minneapolis, MN), anti-cyclooxygenase-2 (COX-2; 1:1,000; Cayman Chemical, Ann Arbor, MI), anti-lipoxygenase 5 (LOX5) (1:1,000; Cayman Chemical), anti-nuclear factor-kappaB (NFκB; 1:1,000; Santa Cruz Bio, Santa Cruz, CA), anti-I kappaB-alpha (1:1,000; Santa Cruz), anti-phospho-I kappaB-alpha (1:1,000; Santa Cruz), or anti-β-actin (1:2,000; Abcam, Cambridge, MA) antibodies for 16 h at 4 °C, after which they were incubated with the appropriate peroxidase-linked secondary antibody for 1 h at room temperature. Chemiluminescence signals were developed with the SuperSignal West Femto Chemiluminescent Substrate (Pierce), and the signals were detected with a digital imaging system (IS4000R; Kodak, New Haven, CT). The intensities of the protein bands were determined using ImageJ 1.32j software.

### Statistical analyses

Statistical analyses were performed by using GraphPad Prism 5.0 software (GraphPad Software Inc., La Jolla, CA). The quantitative data are expressed as mean±standard deviation (SD) or standard error of the mean (SEM) for each group. An unpaired Student *t* test was performed for the means of two unmatched groups; for three or more groups, one-way ANOVA was used to compare each pair of the test groups.

## Results

### The cytoprotective effect of caffeic acid phenethyl ester in 661W cells

**Figure 1:**
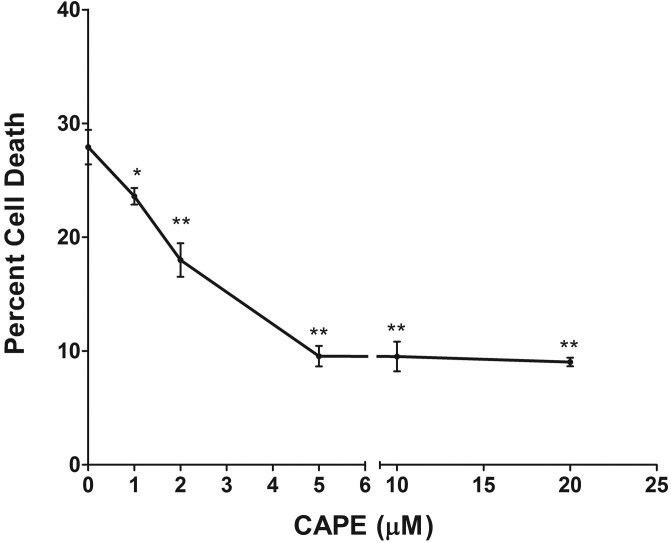
Caffeic acid phenethyl ester (CAPE) protects 661W cells from oxidant-induced cell death. 661W cells were pretreated in situ with 1 to 20 μM CAPE for 3 h. After thorough washing, cells were exposed to 1 mM H_2_O_2_ for 6 h. Cell death was then measured by analyzing the release of lactate dehydrogenase (LDH; n=4 plate × 4 replication assay). (*: p<0.01; **: p<0.001; by one way ANOVA)

The 661W cells were pretreated with varied doses of CAPE (from 1 to 20 µM) for 3 h, washed the cells, waited 3 h, and then challenged the cells with 1 mM H_2_O_2_ for 6 h. This oxidant challenge caused 27% cell death. Pretreatment with CAPE reduced the cell death in a dose-dependent manner up to 5 μM (Figure 1). The cells were then harvested and extracted the mRNA and proteins. An analysis was conducted for the expression of the genes involved in oxidative stress and the proteins involved in apoptotic and protective signaling.

### Gene expression in 661W cells

**Figure 2:**
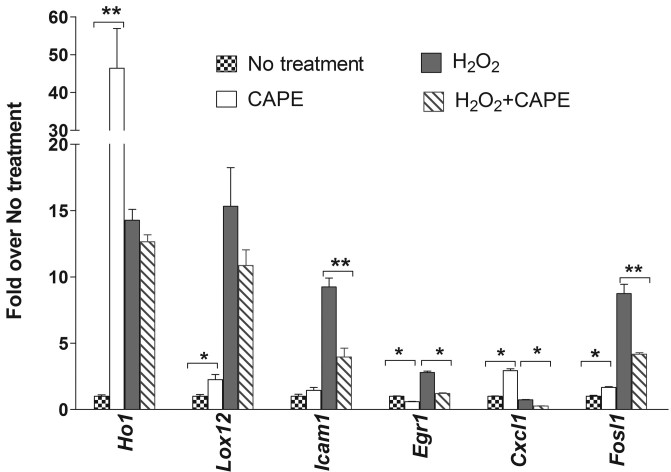
Gene expression in 661W cells was measured by quantitative reverse transcriptase PCR (qRT–PCR). Gene expression was analyzed with the comparative *C*_t_ value method after normalizing against the housekeeping gene, *Rpl19*. Expression values (±SD) are presented against fold change over no-treatment value, which was set to 1.0 (n=3 samples ×3 replication assay per sample). No treatment: no treatment with CAPE or H_2_O_2_; H_2_O_2_:treated with 1 mM of H_2_O_2_ for 6 h; caffeic acid phenethyl ester (CAPE): treated with 5 μM CAPE for 3 h; H_2_O_2_ + CAPE: treated with 5 μM CAPE for 3 h, and then treated with 1 mM of H_2_O_2_ for 6 h (*: p<0.05; **: p<0.01, by the Student *t* test).

Expression of a series of genes that includes the antioxidant pathway and survival pathway were analyzed from the CAPE-treated 661W cells by using qRT–PCR. The expression data were analyzed with the comparative *C*_t_ value method after normalizing against the housekeeping gene, 60S Ribosomal protein L19 (*Rpl19*). Compared to the cells with no treatment, cells pretreated with CAPE for 3 h had significantly induced expression of the heme oxygenase 1 (*Ho1*) gene (Figure 2, 30 fold, p<0.01). These cells also had significantly induced expression of FOS-like antigen 1 (*Fosl1*) by 70% (p<0.05), chemokine (C-X-C motif) ligand 1 (*Cxcl1*) by threefold (p<0.01), and lipoxygenase 12 *(Lox12*; p<0.05); however, early growth response 1 (*Egr1*) expression decreased significantly by twofold (p<0.01).

Treatment with 1 mM H_2_O_2_ for 6 h slightly induced the expression of *Ho1*, intercellular adhesion molecule 1 (*Icam1*), *Lox12*, *Egr1*, and apoptotic gene *Fosl1* (Figure 2). However, pretreatment of CAPE significantly reduced the expression of the *Icam1*, *Egr1*, *Cxcl1*, and *Fosl1* genes (Figure 2).

### Protein expression of heme oxygenase 1, cyclooxygenase-2, and IkappaB-alpha in 661W cells

**Figure 3:**
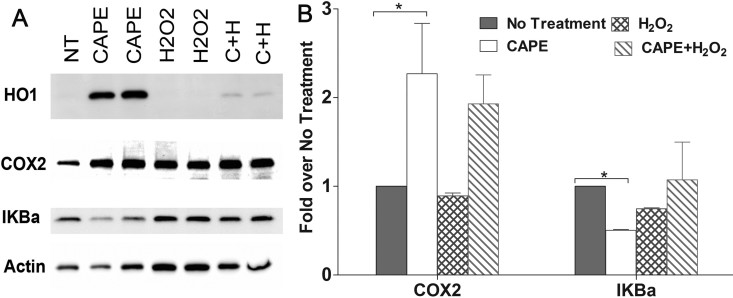
Expression and quantification of selected proteins in 661W cells treated with caffeic acid phenethyl ester (CAPE) and H_2_O_2_. **A**: Expression and quantification of heme oxygenase 1 (HO-1), cyclooxygenase 2 (COX-2), and IκBα proteins in 661W cells was measured by western blot analysis. Proteins were extracted and subjected to western blotting with anti-HO-1, anti-COX-2, and anti-IκBα antibodies. Lane 1(NT): no treatment; lanes 2 and 3 (caffeic acid phenethyl ester [CAPE]): CAPE treated; lanes 4 and 5 (H_2_O_2_): H_2_O_2_ treated; lanes 6 and 7 (C+H): pretreated with CAPE, then with H_2_O_2_. **B**: Quantification of COX-2 and IκBα in 661W cells with western blotting. Quantification of COX-2 and IκBα was obtained with densitometric analysis, and normalized with β-actin. (n=3–6; *: p<0.05, by the Student *t* test).

The expression of select proteins involved in cellular protective and inflammatory signaling was assayed. As shown by the gene expression studies, treatment of CAPE alone induced HO-1 protein expression to a significant level (Figure 3A), and interestingly CAPE action on HO-1 protein persisted even after 6 h of treatment with 1 mM H_2_O_2_ (Figure 3A: C+H). In addition, the level of COX-2, an inducible enzyme that acts as a dioxygenase, a peroxidase, and a potent mediator of inflammation, increased (Figure 3A). Quantification analysis showed the COX-2 protein expression increased about twofold upon treatment with CAPE (p<0.05, Figure 3B), and remained high even when treated with H_2_O_2_.

On the other hand, IκBα expression decreased with CAPE treatment but returned to normal levels when treated with H_2_O_2_ (Figure 3A,B). With a phosphospecific antibody, no phosphorylation was detected in this protein in any of the treatment groups (data not shown), indicating NFκB signaling is probably suppressed or not involved in this scenario. These results support the notion that CAPE could activate the cellular antioxidative defense mechanism by activating the related genes and proteins in the retina-derived 661W cells.

### Functional evaluation with electroretinography and morphologic evaluation with quantitative histology

**Figure 4:**
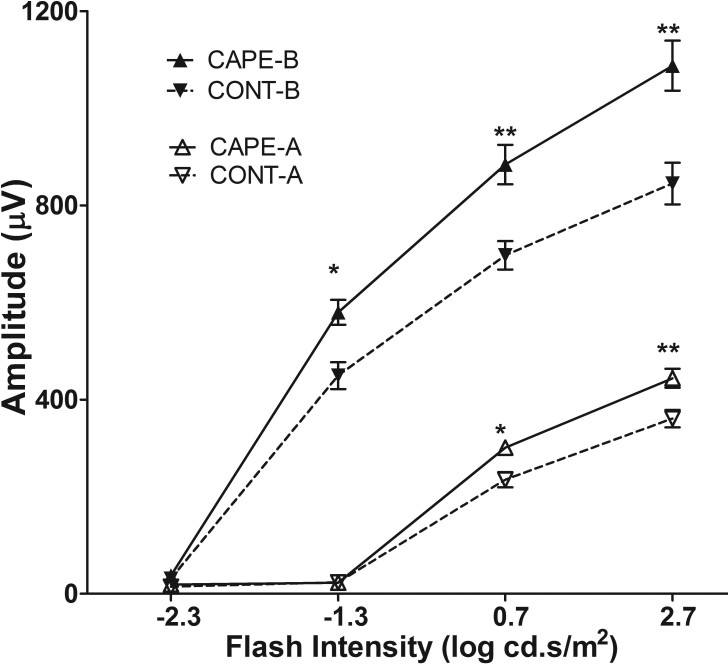
Retinal function measured by electroretinography (ERG) in rats fed for three weeks under cyclic dim (50 lx) light. Scotopic ERG-A (absolute value) and B wave amplitude were analyzed from the rats fed for three weeks with CAPE and reared under dim cyclic light. CAPE-A: A wave from rats fed by CAPE diet; CONT-A: A wave from rats fed by control diet; CAPE-B: B wave from rats fed by CAPE diet; and CONT-B: B wave from rats fed by control diet. (n=8/ group; *: p<0.01, **: p<0.001, with the Student *t* test).

**Figure 5:**
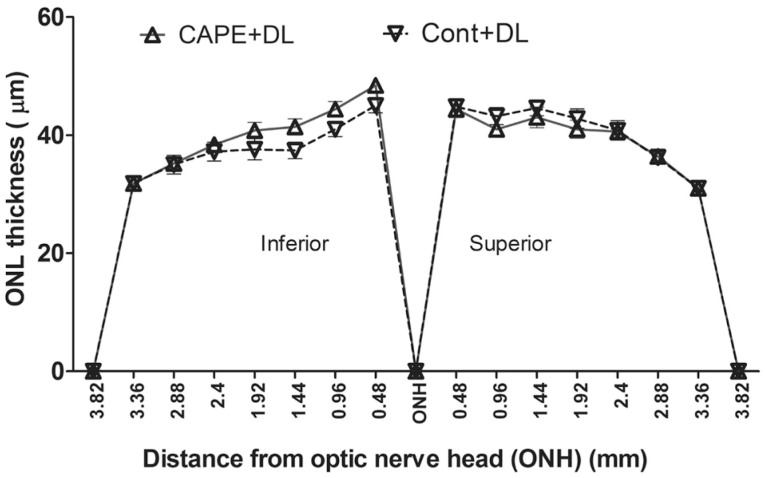
Retina outer nuclear layer (ONL) thickness from rats fed for three weeks under cyclic dim light. The ONL thickness measured in μM at each defined area from superior to inferior through optic nerve. There is no significant difference in ONL thickness of caffeic acid phenethyl ester (CAPE)-fed retina and control-fed retina (p>0.05, with the Student *t* test; n=8). Cont+DL: control diet under cyclic dim light; CAPE+DL: CAPE diet under cyclic dim light.

To understand CAPE’s role in the retina in vivo, the SD rats’ diet was supplemented with CAPE, and then the rats were subjected to either acute intense light stress or chronic cyclic dim/bright light exposure and their retinas analyzed from structural, functional, and biochemical standpoints. Feeding rats with a 0.02% CAPE diet for two weeks did not protect the retinas from intense-light-induced damage (2,700 lux for 6 h, data not shown). Interestingly, compared to the rats fed with the control diet, the CAPE-fed rats maintained in dim light (DL; 50 lux for 3 weeks, 7 AM to 7 PM) had significantly higher ERG scotopic A and B wave amplitudes (Figure 4); however, no significant difference was observed in the photopic ERG (data not shown) or in the photoreceptor cell numbers (measured by ONL thickness; Figure 5).

**Figure 6:**
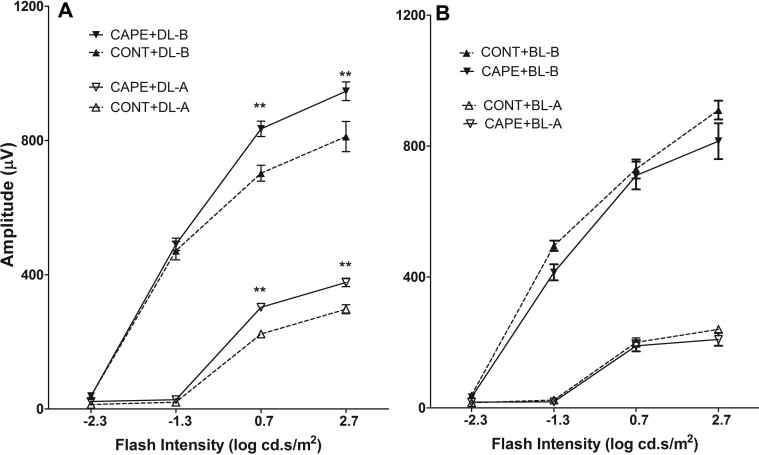
Retinal function measured by electroretinography (ERG) in rats fed for eight weeks and maintained under cyclic dim or bright light. **A**: Scotopic ERG-A and B wave amplitudes of rats fed for eight weeks and maintained in cyclic dim light (DL, 50 lux; n=12; one-way ANOVA). **B**: Scotopic ERG-A and B wave amplitudes of rats fed for eight weeks and maintained in cyclic bright light (BL, 200 lux; n=12; one-way ANOVA). CONT+DL: Rats fed with control diet and maintained under cyclic dim (50 lux) light for eight weeks; CAPE+DL: Rats fed with CAPE diet and maintained under cyclic dim (50 lux) light for eight weeks; CONT+BL: Rats fed with control diet and maintained under cyclic bright (200 lux) light for eight weeks; CAPE+BL: Rats fed with CAPE diet and maintained under cyclic bright (200 lux) light for eight weeks. (n=12; *: p<0.01, **: p<0.001, one-way ANOVA).

**Figure 7:**
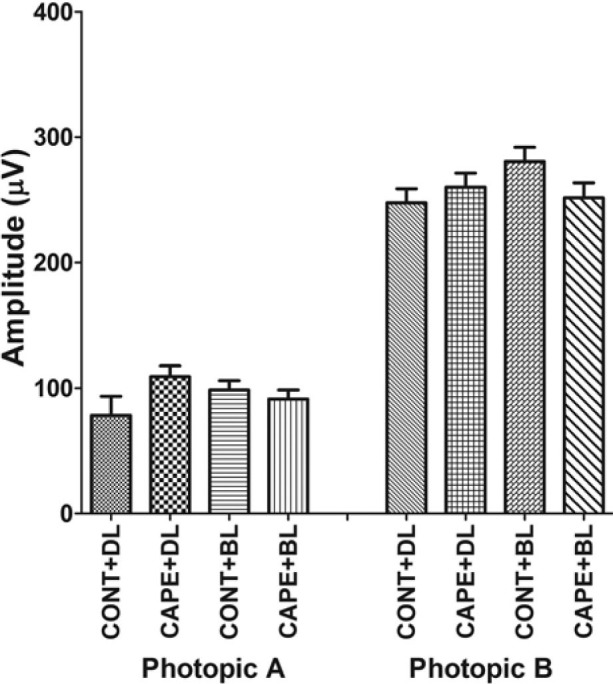
Cone photoreceptor function was measured by electroretinography (ERG) after eight weeks of feeding with control and caffeic acid phenethyl ester (CAPE) diet. Cone photoreceptor function was measured with single-flash photopic ERG with a flash stimulus of 3.7 log cd.s/m^2^ that was presented to dilated, light-adapted (5 min at 2.0 log cd.s/m^2^) rats. There is no significant difference (n=12/group) observed in cone function among the rats fed with CAPE and reared in different light conditions. Cont+DL: control diet under cyclic dim light; CAPE+DL: CAPE diet under cyclic dim light; Cont+BL: control diet under cyclic bright light; CAPE+BL: CAPE diet under cyclic bright light.

**Figure 8:**
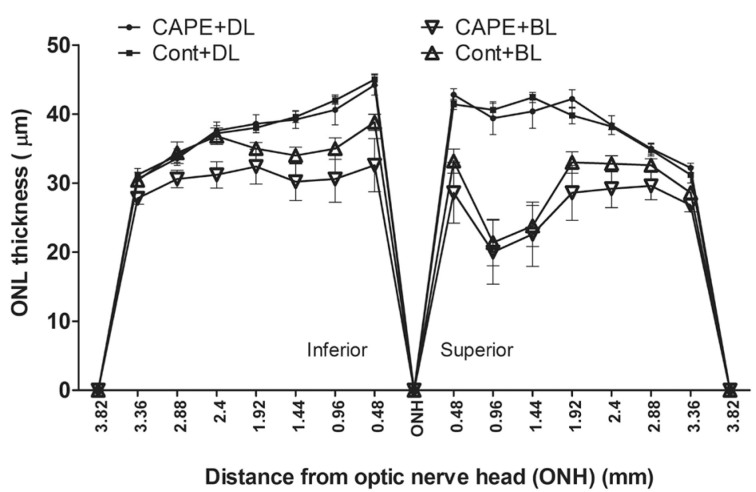
Measurement of retina outer nuclear layer (ONL) thickness from rats fed for eight weeks with control and caffeic acid phenethyl ester (CAPE) diet. The ONL thickness measured in μM at each defined area from the superior to the inferior through the optic nerve. There is no significant difference between the ONL thickness of the caffeic acid phenethyl ester (CAPE)-fed retina and the control-fed retina either from cyclic dim light (DL) or from cyclic bright light (BL) (n=12/group; p>0.05, with the Student *t* test). However, bright light rearing reduced the ONL thickness significantly from dim light rearing. Cont+DL: control diet under cyclic dim light; CAPE+DL: CAPE diet under cyclic dim light; Cont+BL: control diet under cyclic bright light; CAPE+BL: CAPE diet under cyclic bright light.

To further confirm the enhanced ERG responses from rats fed with CAPE in cyclic dim light, the rat number was increased from eight to 12 in each group, and the duration of the feeding period was increased from three weeks to eight weeks in cyclic dim light (50 lux). Another group that was fed for eight weeks with CAPE and exposed to bright cyclic light (200 lux) was established to determine whether CAPE can protect the retina from cyclic-bright-light-induced, chronic photoreceptor loss. Similar to the three-week feeding results (Figure 4), the eight-week CAPE feeding resulted in significantly higher ERG responses in the dim-reared rats (scotopic A and B; p<0.01; Figure 6A). However, no significant difference was observed in the ERG responses between the CAPE- and control-fed rats maintained in bright cyclic light (200 lux) for eight weeks (Figure 6B). The cone response as measured with photopic ERG, and the ONL thicknesses were still not significantly different between the CAPE- and the control-fed rats under cyclic dim or bright light (Figure 7 and Figure 8). The retinas were further examined using biochemical and molecular analyses to understand the effect of dietary CAPE on retinal tissue, with a focus on oxidative and inflammatory markers.

### Gene and protein expression in the retina

The expression of a series of genes known to be involved in oxidative stress and proinflammatory processes, including *Ho1*, *Icam1*, *Cox2*, *Catalase*, *Cxcl1*, *Lox5*, *Lox12*, *Ccl2*, *Fosl*, interleukin-6 (*IL6*), *c-Fos,* thioredoxin 1 (*Thx1*), *Nfkb,* and glial fibrillary acidic protein (*Gfap*), were measured in the retinas from CAPE-fed rats maintained in either dim or bright cyclic light for eight weeks. All expression values were normalized to the housekeeping gene, *Rpl19*.

**Figure 9:**
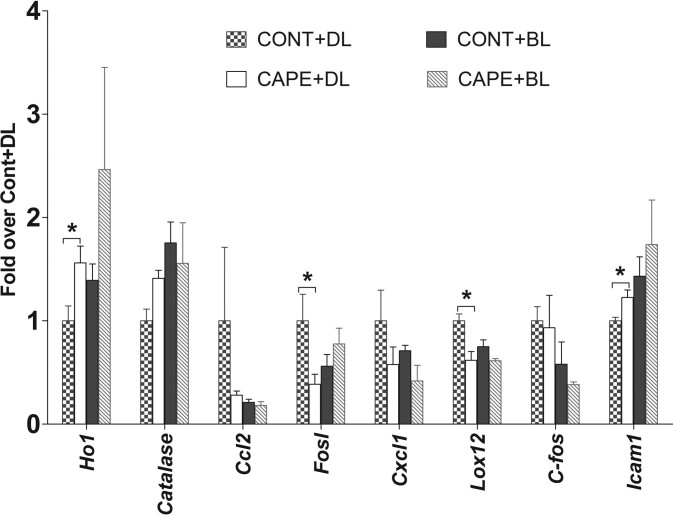
Expression of selected genes in rat retina as measured by quantitative reverse transcriptase PCR (qRT–PCR). Retinas were harvested from rats maintained under cyclic dim or bright light exposure for eight weeks. Expression values were normalized with the housekeeping gene, *Rpl19*. Expression values (±SD) are presented as fold change over the CONT+DL value, which was set to 1.0 (n=4 samples). CONT+DL: control diet under cyclic dim light; caffeic acid phenethyl ester (CAPE)+DL: CAPE diet under cyclic dim light; CONT+BL: control diet under cyclic bright light; CAPE+BL: CAPE diet under cyclic bright light. (*: p<0.05, with the Student *t* test).

Compared to the retinas from rats fed with the control diet in DL, the retinas from the CAPE-fed rats in DL had significantly higher expression levels of *Ho1* and *Icam1* (p<0.05) and significantly lower expression levels of *Fosl1* (proapoptotic) and *Lox12* (proinflammatory) (2.5-fold and one-fold, respectively, p<0.05 for each, Figure 9). However, no significant differences were found in the expression of *Cox2*, *catalase*, *Cxcl1*, *Lox5*, *Ccl2*, *IL6*, *C-fos*, *Thx1*, *Nfkb*, and *Gfap*.

**Figure 10:**
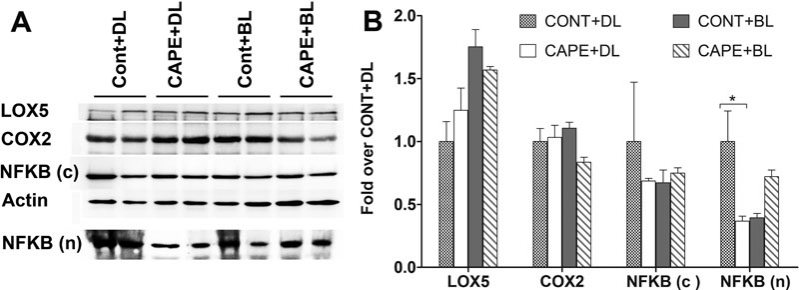
Expression and quantification of selected proteins in the retina from rats fed for eight weeks with control and caffeic acid phenethyl ester (CAPE) diet. Retinas were harvested from CAPE- and control diet-fed rats maintained under cyclic dim or bright light exposure for eight weeks. **A**: Retinal whole cell proteins were extracted and subjected to western blotting with anti-LOX5, and anti-COX-2 antibodies, and the cytoplasmic and nuclear fraction separately with anti-NFκB antibodies. **B**: Quantification of LOX5, COX-2, and NFκB (n) expression in nuclei and NFκB (c) in cytosol from the retinas was obtained with densitometric analysis, and normalized by β-actin, (n=4). Cont+DL: control diet under cyclic dim light; CAPE+DL: CAPE diet under cyclic dim light; Cont+BL: control diet under cyclic bright light; CAPE+BL: CAPE diet under cyclic bright light. (*: p<0.05, with the Student *t* test).

In addition, the NFκB protein level in the nuclear fraction was significantly lower in the retinas from CAPE-fed rats under cyclic dim light than in the retinas from rats fed the control diet, but the protein levels of LOX5, COX-2, and cytosolic NFκB were not different between the two groups (Figure 10A,B).

However, no significant differences were detected in the expression levels of the *Ho1*, *Icam1*, *Cox2*, *catalase*, *Cxcl1*, *Lox5*, *Lox12*, *Ccl2*, *Fosl*, *IL6*, *C-fos*, *Thx1*, *Nfkb*, and *Gfap* genes and the LOX5, COX-2, and NFκB proteins in nuclear and cytoplasmic fractions from the retinas of rats maintained under cyclic BL for eight weeks (Figure 9, Figure 10A,B).

### Fatty acid analysis

Since CAPE can inhibit lipid peroxidation [47] and lipoxygenase activities [48], it may affect fatty acid metabolism in the retina. The fatty acid profile in the retinas from rats fed with the CAPE or control diet for eight weeks that were maintained either in cyclic dim or bright light were analyzed. Compared to the control-fed rats, the CAPE-fed rats maintained in dim cyclic light had significantly higher relative mole percentages of the fatty acids 14:0, 18:1, and 18:3n3 and the ratio of n6/n3-fatty acids (p<0.05), and significantly lower the fatty acids 18:0, 20:2n6, 20:4n6, 22:0, 22:1, 22:4n6, 22:6n3 (docosahexaenoic acid), 24:0, 24:1, and 24:6n3 (p<0.05) (Table 2). However, for the rats maintained under bright cyclic light no changes were detected (p>0.05), except for significantly higher relative mole percentages of 22:1, 22:5n3, and 24:1 fatty acids in the CAPE-fed rats than in the control-fed rats (p<0.05, Table 2).

**Table 2:** Relative mole percentage (±SD) of fatty acid composition from rat retina fed by CAPE or control diet under dim or bright cyclic light rearing.

	**Under dim light (DL)**			**Under bright light (BL)**		
**Fatty acid**	**CONT+DL**	**CAPE+DL**	**p-value**	**CONT+BL**	**CAPE+BL**	**p-value**
14:0	0.96± 0.06	1.68±0.28	<0.05	0.94±0.11	0.84±0.04	0.4
18:0	17.97±0.29	13.28±1.49	<0.05	18.64±1.57	20.10±0.09	0.3
18:1	15.84±0.28	19.37±1.49	<0.05	16.38±1.08	15.35±0.12	0.3
18:2n6	9.05±0.51	13.27±2.05	0.07	8.46±1.44	6.80±0.08	0.22
18:3n6	0.08±0.00	0.09±0.01	0.39	0.08±0.01	0.07±0.00	0.45
18:3n3	0.60±0.04	1.13±0.19	<0.05	0.53±0.16	0.35±0.01	0.22
20:2n6	0.47±0.03	0.35±0.02	<0.05	0.43±0.06	0.49±0.02	0.39
20:4n6	9.71±0.48	7.58±0.83	0.06	10.61±0.68	11.43±0.46	0.37
22:0	0.30±0.01	0.22±0.02	<0.05	0.35±0.04	0.40±0.01	0.23
22:1	0.03±0.00	0.02±0.00	<0.05	0.03±0.00	0.05±0.00	<0.05
22:4n6	0.65±0.09	0.29±0.04	<0.05	0.35±0.02	0.35±0.02	0.97
22:5n3	0.67±0.06	0.56±0.07	0.27	0.55±0.01	0.72±0.02	<0.05
22:6n3	15.53±0.28	10.38±1.97	<0.05	14.41±1.07	15.00±0.78	0.68
24:0	0.45±0.02	0.32±0.03	<0.05	0.53±0.04	0.58±0.02	0.34
24:1	0.33±0.03	0.23±0.01	<0.05	0.34±0.02	0.40±0.01	<0.05
24:6n3	0.24±0.00	0.16±0.03	<0.05	0.17±0.01	0.16±0.02	0.69
n6/n3	1.16±0.06	1.82±0.26	<0.05	1.27±0.12	1.18±0.09	0.59

## Discussion

The cytoprotective mechanism of CAPE as determined by several studies is multioriented. CAPE is a potent and specific inhibitor for suppressing NFκB activation [49], lipid peroxidation [47], lipoxygenase activities [48], protein tyrosine kinase activity [50], and ornithine decarboxylase activity [51]. We found that CAPE protected retina-derived 661W cells against H_2_O_2_-induced cell death in vitro, which was accompanied by changes in the expression of a series of antioxidant genes and proteins. Supplementation with CAPE in the diet can also modulate the expression of a series of antioxidant genes and proteins in albino rat retinas.

Although the molecular mechanism of the cytoprotective role of CAPE in retinal cells is not known, our data suggest that CAPE may act through inhibiting NFκB [49]. Activation of NFκB, known to act as a master regulator for the expression of many genes, may induce the apoptotic process. In the cytosol, NFκB remains inactive by forming a complex with inhibitory proteins of IκBα. In response to stimuli, IκBα kinases (IκKs) mediate IκBα phosphorylation, the dissociation of the NFκB inhibitory complex, and the activation of NFκB, which then translocates to the nucleus [52,53]. Activation and nuclear localization of the transcription factor NFκB are reportedly found in light-stressed retinas from mice and may play a role in light-induced photoreceptor degeneration [54,55]. In our study, we observed not only reduced IκBα expression in the CAPE-treated 661W cells (Figure 3A,B) but also a significant decrease in the amount of NFκB translocated to the nucleus in the retinas of CAPE-fed rats (Figure 10A). This observation is consistent with observations from previous studies; inhibiting NFκB activation by CAPE may be a mechanism of CAPE-mediated anti-inflammatory and antioxidation effects [23].

Emerging evidence suggests that CAPE may also act through inducing the heme oxygenase 1 system to manifest antioxidant characteristics [56-59]. HO-1 is a ubiquitous and redox-sensitive, inducible stress protein [60]. HO-1 catalyzes the degradation of heme to generate carbon monoxide, free ferrous iron, and biliverdin [61]. Further data supported a pivotal role for HO-1 in the resolution of acute inflammatory states [62], as well as a role in protecting against oxidative stress [63,64]. We found dramatic increases in the gene and protein expression of HO-1 in the 661W cells treated with CAPE for 3 h (Figure 2 and Figure 3A**)**, and increased gene expression in the retinas of rats fed with CAPE and maintained under cyclic dim light (Figure 9). These data further support the action of CAPE as an inducer of HO-1 to represent an efficient antioxidant system and a potential pharmacological target for a variety of oxidant- and inflammatory-mediated diseases, including brain aging and neurodegenerative disorders [65-69].

We also found CAPE treatment downregulated the oxidant-induced expression of *Egr1* and *Icam1* in 661W cells. EGR1, a Zn^2+^ finger-containing transcription factor, is known as a mediator of oxidative stress-induced tissue damage and controls the expression of many inflammatory genes, including *Icam1* [70,71]. The CAPE-mediated decrease in *Egr1* and *Icam1* expression levels in 661W cells indicates its anti-inflammatory activity may also be mediated through the EGR1 transcription factor. An induction of COX-2 was recorded in the CAPE-treated 661W cells and in the cells treated with CAPE and H_2_O_2_ (Figure 3B). However, in the retina treated with CAPE under either dim or bright light the COX-2 level did not change. It could be a hormetic response in the cultured 661W cells, in which CAPE induced slight inflammatory stress to the cells, which in turn activated a cellular protective system, such as induction of HO-1 gene expression. Further studies are needed to understand this response.

Interestingly, we found that dietary supplementation of CAPE enhanced the amplitudes of ERG A and B wave responses (Figure 4, Figure 6A) in dim-reared rats, which is a novel observation. However, how CAPE influenced the scotopic ERG responses is not clear. Along with the enhanced ERG amplitudes, we found significant changes in a series of fatty acids in the retinas from the CAPE-fed rats. The retinas from CAPE-fed and cyclic dim light-maintained rats had higher molar ratios of 14:0, 18:1, and 18:3n3 fatty acids and a lower content of 22:6n3 and 18:0, 20:2n6, 20:4n6, 22:0, 22:1, 22:4n6, 24:0, 24:1, and 24:6n3 fatty acids (Table 2). We speculate that altered fatty acid composition in the retinas of the CAPE-fed rats probably contributed to the higher ERG responses. In the 1970s, Anderson et al. found that fatty acid composition in rod outer segment membranes is an important determinant for optimal retinal function in rodents and alterations are associated with modifications of retinal function as measured with ERG [72,73]. Later, many studies reported that alterations in fatty acids, especially docosahexaenoic acid content and altered n6/n3, are associated with altered phototransduction efficiency and thus ERG responses [74-78]. Further, the rats maintained under bright (200 lux) cyclic light for eight weeks had no significant changes in the fatty acid levels or the n6/n3 ratio and no significant change in ERG responses, which provides further support to our hypothesis that the altered fatty acid composition of the retina may be associated with altered ERG responses (Table 2 and Figure 6A,B). Previous studies reported that natural compounds, such as ginseng extract and saffron, could alter (enhance) the ERG responses in animal models and in humans by some unknown mechanism [79,80].

Although CAPE changed the fatty acid profile in the retina, how CAPE affects the fatty acid profile in the retina is unclear. Since CAPE is a potent and specific inhibitor of lipid peroxidation [48] and can inhibit lipoxygenase 5 [47] and lipoxygenase 15 [81], we speculate that the modulated lipoxygenase activity by CAPE in the retina might partially contribute to the altered fatty acid composition. Further experiments are needed to clarify these probable pathways.

Finally, we did not find that CAPE protected the retina from damage induced by acute bright light (2700 lux for 6 h) and chronic bright light (200 lux for 8 weeks). This might be due to the low amount of CAPE (0.02%) present in the diet, such that the amount that was absorbed and reached the retina might not be enough to protect against strong insults such as 2,700 lux light for 6 h or 200 lux cyclic light for eight weeks. The bioavailability of CAPE through diet and its transportation to the retina, therefore, needs further investigation.

In conclusion, we found CAPE activated the antioxidant gene expression pathway in 661W cells and in rat retinas, and enhanced the scotopic ERG A/B wave amplitude and altered fatty acid profile in the retinas of rats maintained under cyclic dim light. These data support further investigation into the potential therapeutic benefits of CAPE in protecting the retina from oxidative and/or inflammatory stress.
